# Readiness for sugar sweetened beverage taxation in sub-Saharan Africa

**DOI:** 10.1080/16549716.2021.1917801

**Published:** 2021-04-20

**Authors:** Ntombizodwa Ndlovu, Boyd Swinburn

**Affiliations:** a School of Public Health, Faculty of Health Sciences, University of the Witwatersrand, Johannesburg, South Africa; b School of Population Health, University of Auckland, Auckland, New Zealand

The picture of Africa as a starving continent is rapidly being replaced by that of an overweight continent. While undernutrition is still high in Sub-Saharan Africa (SSA), obesity and nutrition-related non-communicable diseases (NR-NCDs) are rising rapidly [1]. It is projected that NCDs will be the leading cause of death in Africa by 2030 [2]. SSA finds herself in a nutrition transition moving from the consumption of healthy, traditional, unrefined foods, to cheap, unhealthy, aggressively marketed, ultra-processed foods. Western-style food systems are infiltrating across the continent [3]. In some African cultures, the situation is exacerbated by sociocultural beliefs that associate obesity with material wealth and social status [3].

In Africa, NR-NCDs add to the heavy health and economic burdens in a region with high prevalence of communicable diseases and poverty. Yet efforts to tackle obesity and prevent NCDs are being hampered by scarce healthcare resources, limited government spending, and restricted foreign investments and aid. The World Health Organization (WHO) recommendation of a 20% tax on sugar-sweetened beverages (SSBs) requires urgent policy attention by governments in SSA [4].

Increasingly, low- and middle-income countries are considering substantial SSB taxation to address NR-NCDs [5], with the revenue raised being used for disease treatment and prevention. There is considerable international evidence on the impact of SSB taxes – the challenge is implementation.

This Special Issue of Global Health Action comprises a collection of papers from seven SSA countries which sought to identify opportunities to implement or strengthen SSB taxation policy in the region. Thow et al. [6] presented the study design for the policy landscape analysis of SSB taxation across the seven countries in a paper published earlier. The methodology was developed by researchers in the participating countries from eastern (Kenya, Rwanda, Tanzania, Uganda) and southern Africa (Botswana, Namibia, Zambia) and also South Africa and Australia. The systematic methodological approach was informed by several policy analysis frameworks, recommendations, and learnings from countries, like South Africa, that have implemented SSB taxation. Opportunities for policy change were identified focusing on the problems (evidence and perceptions about the policy problem), policy (existing policies and proposed solutions) and politics (political and institutional contexts).

All five individual country studies presented in this Special Issue investigated some form of excise country-level tax on beverages. In Zambia, local research showed that a 25% tax would significantly reduce SSB consumption and generate revenue for government [7]. However, industry discredited the findings, resulting in the adoption of a weak, 3% excise tax – far below the WHO’s 20% tax recommendation. Botswana did not provide an individual study, but it is noteworthy that the country introduced an SSB excise tax of 2 thebe (cents) per gram of sugar on 1 April 2021 [8].

Socioeconomic factors such as education and literacy, poverty, and unemployment influence food decision-making. Participants in Zambia highlighted perceptions that NR-NCDs are diseases of the wealthy and SSB consumption is a health hazard for the elite, educated and middle class [7]. They also noted the ‘shunning’ of nutritious traditional foods in favour of unhealthy and cheaper ‘high status’ options. In Kenya, it was observed that SSBs cannot be ‘criminalized’ by imposing a tax as was the case for tobacco, because SSBs are ‘food’ [9]. The authors recommended that the public should be provided with adequate information about the adverse health effects of SSBs and sugar consumption. The study in Rwanda highlighted that access to healthy alternative beverages is important as only 57% of the population have access to safe drinking water [10].

There are tensions between health and economic policies. For example, in Uganda, the Ministry of Health promotes taxation on sugar, albeit to generate revenue and not specifically to reduce SSB consumption. Conversely, the Ministry of Finance works to ensure that the country’s confectionary industry remains regionally competitive, and that the SSB industry uses multiple strategies, including corporate social responsibility programmes, to maintain its market and profitability [11]. In Rwanda, major health-sector reforms have resulted in improved health outcomes over the past two decades [10]. However, current employment and investment agendas to bolster the sugar industry do not align with public health efforts to address unhealthy diets and obesity.

Unlike its neighbour, South Africa, Namibia is yet to introduce SSB taxation for NR-NCD prevention [12]. It was suggested that the strong political and trade ties between the countries could be leveraged to launch a regional advocacy case for SSB taxation.

The seven-country comparative papers together draw a regional perspective on the prospects for SSB taxation. Having common methodologies is critical for measuring progress, supporting advocacy efforts, and evaluating policy impacts. Other countries in Africa and elsewhere can use these methods to expand the benchmarking database. This has already occurred with more than 85 institutions in 58 countries using protocols of the new INFORMAS initiative to benchmark food environments [13] and create rich comparative datasets [14].

The paper on political economies of SSB taxes in the seven countries by Thow et al. [15] showed that while NR-NCDs were recognised as a dominant and rising health problem, serious fiscal solutions had not been implemented. If other SSA countries were to follow South Africa in implementing SSB taxation for health purposes, perhaps the domino effect would continue across the region; all countries already have excise tax collection machinery in place. However, the prevailing economic paradigm of sugar cane production and SSB manufacturing as important strategies for economic growth would need to change.

Abdool Karim et al. [16] investigated the legal feasibility of adopting a taxation policy on SSBs in seven SSA countries and concluded that there are minimal legal barriers for governments that wish to change the purpose of their excise taxes from small, revenue generation to substantial, health-promoting taxes.

Internationally, every country that has proposed an SSB tax has battled against the lobbying power of wealthy multinational industry giants such as Coca-Cola and PepsiCo, which target African countries as growth markets. The availability of good data is critical for making the case for policy, as shown in countries like Mexico [17] and the UK [18]. The seven-country paper on relevant data availability by Erse et al. shows that SSA countries are not in a strong position to use local data to argue for SSB taxes [19]. However, the authors’ mapping of the data availability landscape provides important information for the strengthening of monitoring systems.

This Special Issue of Global Health Action on the readiness for sugar-sweetened beverage taxation makes a valuable contribution to the literature on how low- and middle-income countries could use this effective, evidence-based strategy to respond to under-recognized, but increasingly prevalent, epidemics of obesity and NR-NCDs.

## References

[1] NCD Risk Factor Collaboration. Worldwide trends in body-mass index, underweight, overweight, and obesity from 1975 to 2016: a pooled analysis of 2416 population-based measurement studies in 128.9 million children, adolescents, and adults. Lancet. 2017;390:2627–3. 29029897 10.1016/S0140-6736(17)32129-3 PMC5735219

[2] Marquez P, Farrington J. The challenge of non-communicable diseases and road traffic injuries in sub-Saharan Africa: an overview. Washington (DC): World Bank; 2013.

[3] BeLue R, Okoror TA, Iwelunmor J, et al. An overview of cardiovascular risk factor burden in Sub-Saharan African countries: a socio-cultural perspective. Global Health. 2009;5:10. 19772644 10.1186/1744-8603-5-10 PMC2759909

[4] World Health Organization (WHO). Fiscal policies for diet and the prevention of non-communicable diseases. WHO; 2015.

[5] Bridge G, Lomazzi M, Bedi R. Implementation of a sugar-sweetened beverage tax in low- and middle-income countries: recommendations for policymakers. J Public Health Policy. 2020;41:84–97. 31740712 10.1057/s41271-019-00196-z

[6] Thow AM, Erzse A, Asiki G, et al. Study design: policy landscape analysis for sugar-sweetened beverage taxation in seven sub-Saharan African countries. Glob Health Action. 2021;14:1856469. 33475471 10.1080/16549716.2020.1856469 PMC7833028

[7] Mukanu MM, Abdool Karim S, Hofman K, et al. Nutrition-related non-communicable diseases and sugar sweetened beverage policies: a landscape analysis in Zambia. Glob Health Action. 2021. 10.1080/16549716.2021.1872172 PMC8079008 33876714

[8] Deloitte tax@hand [Internet]. New York (NY): Deloitte Global Services Limited. Tax highlights of 2021/22 budget: 2021 10 2 [cited 2021 48]. Available from: https://www.taxathand.com/article/16146/Botswana/2021/Tax-highlights-of-202122-budget?id=gx:2em:3na:4wta_2021_02_12:5eng:6tax&sfid=7011O0000038lKVQAY#:~:text=The%20introduction%20of%20a%20sugar,beverages%20such%20as%20fizzy%20drinks

[9] Wanjohi MN, Thow AM, Abdool Karim S, et al. Nutrition-related non-communicable disease and sugar-sweetened beverage policies: a landscape analysis in Kenya. Glob Health Action. 2021. 10.1080/16549716.2021.1902659 PMC8079030 33874855

[10] Ruhara CM, Abdool Karim S, Erzse A, et al. Strengthening prevention of nutrition-related non-communicable diseases through sugar-sweetened beverages tax in Rwanda: a policy landscape analysis. Glob Health Action. 2021. 10.1080/16549716.2021.1883911 PMC8079049 33876706

[11] Ahaibwe G, Abdool Karim S, Thow AM, et al. Barriers to, and facilitators of, the adoption of a sugar sweetened beverage tax to prevent non-communicable diseases in Uganda: a policy landscape analysis. Glob Health Action. 2021. 10.1080/16549716.2021.1892307 PMC8078933 33874854

[12] Amukugo HJ, Abdool Karim S, Thow AM, et al. Barriers to and facilitators of the adoption of a sugar sweetened beverage tax to prevent non-communicable diseases in Namibia: a policy landscape analysis. Glob Health Action. 2021. 10.1080/16549716.2021.1903213 PMC8079035 33876708

[13] Swinburn B, Sacks G, Vandevijvere S, et al. INFORMAS (International network for food and obesity/non-communicable diseases research, monitoring and action support): overview and key principles. Obes Rev. 2013;14:1–12. 10.1111/obr.12087 24074206

[14] Sacks G, Kwon J, Vandevijvere S, et al. Benchmarking as a public health strategy for creating healthy food environments: an evaluation of the INFORMAS initiative (2012-2020). Annu Rev Public Health. 2021;42:345–362. 33351647 10.1146/annurev-publhealth-100919-114442

[15] Thow AM, Abdool Karim S, Mukanu MM, et al. The political economy of sugar-sweetened beverage taxation: an analysis from seven countries in sub-Saharan Africa. Glob Health Action. 2021. 10.1080/16549716.2021.1909267 PMC8078959 33877032

[16] Abdool Karim S, Erzse A, Thow AM, et al. The legal feasibility of adopting a sugar-sweetened beverage in seven Sub-Saharan countries. Glob Health Action. 2021. 10.1080/16549716.2021.1884358 PMC8078924 33876700

[17] Colchero MA, Rivera-Dommarco J, Popkin BM, et al. In Mexico, evidence of sustained consumer response two years after implementing a sugar-sweetened beverage tax. Health Affairs. 2017;36:564–571. 28228484 10.1377/hlthaff.2016.1231 PMC5442881

[18] Bandy LK, Scarborough P, Harrington RA, et al. Reductions in sugar sales from soft drinks in the UK from 2015 to 2018. BMC Med. 2020;18:20. 31931800 10.1186/s12916-019-1477-4 PMC6956503

[19] Erzse A, Thow AM, Ahaibwe G, et al. The data availability landscape in seven Sub-Saharan African countries and its role in strengthening sugar-sweetened beverage taxation. Glob Health Action. 2021. 10.1080/16549716.2020.1871189 PMC8079050 33876702

